# From sedation to stimulation: anecdotal case reports of enhanced sexual arousal in men and women taking diphenhydramine hydrochloride

**DOI:** 10.1093/sexmed/qfag011

**Published:** 2026-03-23

**Authors:** Melitzsa Pascale Regis, James G Pfaus

**Affiliations:** Health Sciences Program, Broward College, Fort Lauderdale, FL 33301, United States; Department of Psychology and Life Sciences, Charles University, Prague 18200, Czech Republic; Center for Sexual Health and Interventions, Czech National Institute of Mental Health, Klecany 25067, Czech Republic

**Keywords:** antihistamine, sexual arousal, orgasm, disinhibition

## Abstract

**Introduction:**

Unexpected enhancement of sexual arousal, desire, and orgasm following diphenhydramine (DPH) ingestion has emerged in anecdotal reports across online platforms.

**Aims:**

To examine whether there are common effects of DPH on the sexual responses of women and men who respond to DPH with excitation rather than sedation.

**Methods:**

Qualitative case data were gathered from surveys of men and women on a DPH Reddit site to examine the consistency, intensity, and subjective nature of DPH effects on sexual arousal, desire, and orgasm.

**Results:**

Participants described enhanced sexual arousal, orgasm, increased stimulation sensitivity, and profound emotional release from DPH. Some found the effects stronger than those of prescription medications for sexual arousal disorders.

**Conclusion:**

These observations suggest a paradoxical pharmacological action of DPH, potentially involving dopaminergic and/or histaminergic pathways. While DPH itself is not a recognized therapeutic agent for the treatment of sexual dysfunctions, these unexpected effects may point toward a novel neurochemical mechanism of sexual excitation in some individuals that warrants further investigation.

## Introduction

Antihistamines are widely believed to induce unwanted sexual side-effects.^1^^,^^2^ Because histamine relaxes smooth muscles allowing for genital blood flow during sexual arousal, antihistamines can disrupt this process leading to sexual arousal problems like erectile dysfunction. Antihistamines also reduce mucous secretion that can lead to vaginal dryness. Many first generation H1 receptor antagonists also induce drowsiness, which can decrease sexual desire and blunt genitosensory stimulation.

Diphenhydramine (DPH) is a first-generation ethanolamine derived antihistamine and anticholinergic agent used commercially for allergies and as a sleep aid. It is known to cause drowsiness and dry mouth,^3^ and is listed among antihistamines that cause unwanted sexual side-effects. However, in some individuals, DPH produces a paradoxical excitation.^4^^,^^5^ DPH acts as an inverse agonist at histaminergic H1 receptors, a competitive antagonist at muscarinic receptors, and produces a mild inhibition of the cytochrome P450 (CYP) 2D6 enzyme, where it acts as a competitive substrate. This latter effect may underlie the paradoxical excitation. People that have unique metabolic profiles, in this case, at least three copies of the CYP2D6 gene, are called ultrarapid metabolizers, and it has been proposed that ultrarapid metabolism of DPH by this enzyme turns it into a stimulant.^5^^,^^6^

Informal user accounts largely on Reddit but also on other public forums (eg, drugs-forum.com; health.narkive.com) increasingly describe a stimulatory effect of DPH on sexual function, including enhanced sexual arousal, intensified genitosensory stimulation and orgasm, and emotional catharsis. These descriptions have not been described previously in the clinical literature. Here we report qualitative assessments from nine anonymous individuals (5 men and 4 women) who self-reported unexpected excitatory effects on sexual arousal following DPH ingestion.

## Method

This exploratory analysis employed a qualitative, anonymous survey to collect experiential data from individuals on a Reddit site devoted to individuals that experience excitation rather than sedation from DPH, including enhanced sexual function. The objective was to gather insights that could inform future clinical or pharmacological research on DPH-induced sexual excitation. Male respondents (M1-M5) ranged in age from 20 to 36 (4 from the US, 1 from Germany). Female respondents (F1-F4) ranged in age from 27 to 36 (3 from the US, 1 from Argentina).

### Participant recruitment

Participants were individuals who had previously posted on Reddit about sexual arousal effects related to DPH. They were recruited by personal outreach from the first author, who did not know the identities of any of the participants. Participation was entirely voluntary and anonymous, and respondents were informed that the project was non-clinical and exploratory. All respondents agreed to answer questions about their demographics, medical and non-medical drug use, and the quality of their sexual responses with and without DPH. They were informed that their responses would remain anonymous and be assembled and sent for publication in a scientific journal. Informed consent was implied through completion of the survey. This general method for reporting anonymized data from online sources was approved by the Human Research Ethics Committee of the Czech National Institute of Mental Health (c.j.92/22).

### Data collection

The primary data collection tool was a custom-designed Google Forms survey with 23 structured and semi-structured questions. These included demographic information, context of DPH use, perceived sexual effects, timing, dose, and subjective experience. Open-ended prompts allowed for elaboration on mood, comparisons to other substances (eg, alcohol, cannabis, PDE-5 inhibitors), and mental health background. Responses were downloaded and coded thematically by the first author. No identifying data were included in the analysis. The survey was designed in alignment with standards for exploratory, non-interventional research on sensitive topics.^7^ Although not a validated instrument, the survey is available upon request.

## Cases and common effects

In all cases, the drug was used originally as a sleep aid. Excitatory effects on mood and sexual responses were reported in doses ranging between 50 mg to 500 mg depending on the respondent, and with effectiveness delayed from 30 min to 3 hrs after oral ingestion of commercial tablets or syrup. In most cases, the enhancing effects were mentioned specifically in the context of masturbation to orgasm. The following themes emerged, with notable interconnections across the domains.

### Heightened orgasmic sensation and genital sensitivity

Respondents M1, M3, M4, M5, and F4 described intensified orgasms and heightened genital sensitivity following DPH ingestion. M1 reported more powerful ejaculatory muscle contractions and increased volume, while M4 noted amplified sensitivity even at low doses. M3 described the climax as “physically and emotionally draining.”

### Consistent arousal response at higher doses

Several respondents (notably M2, M3, and F4) identified a dose-dependent pattern in their arousal experiences. The concept of a “fapdose,” a community-coined term indicating a dose that stimulates masturbation, typically referred to doses above 150 mg, where arousal became reliably triggered. M2 described an uncontrollable compulsion to masturbate at doses exceeding 175 mg, while M3 planned sexual activity around the drug’s onset window. F4 also experienced pronounced arousal from initial use onward, implying an underlying pharmacological consistency at moderate to high doses.

### Arousal independent of baseline desire

Respondents who reported having low or suppressed libido, including F1, F4, and M5, still experienced robust arousal under the influence of DPH. F4, who typically experienced inhibited sexual responses from her antidepressants, noted that DPH “overrode” those effects, enabling sustained sexual arousal, desire, and orgasm capability.

### Psychological conditioning and ritualized use

For some respondents, arousal from DPH became behaviorally conditioned over time. M3 explicitly stated that arousal was not initially present, but became predictable through repeated pairing of DPH with masturbation. M2’s language also reflected ritualized usage. This aligns with broader models of reinforcement learning and behavioral conditioning where the drug context itself acquires motivational salience.^8^

### Drug interactions and modulation

Respondents described using DPH in conjunction with other substances, including alcohol, cannabis, opioids, stimulants, and prescription arousal aids like sildenafil and tadalafil. M1 compared DPH favorably to both sildenafil and tadalafil, emphasizing that the latter supported erectile function but lacked the emotional and sensory depth induced by DPH. M4 and F4 noted that cannabis amplified the arousal effects, while alcohol often blunted them.

### Functional impact and emotional cost

Despite enhanced arousal and orgasm quality, four respondents described a longer-term effect that in some cases resembled post-orgasmic illness syndrome. F1 reported next-day fatigue, mild brain fog, and occasional insomnia even after 8+ hours of sleep. M1 experienced intrusive thoughts and visual hallucinations at high doses. M3 reported post-orgasmic emotional depletion and anhedonia lasting through the remainder of the high, and M4 described a consistent “hangover” effect even at low doses. These responses suggest a longer-term rebound in histamine that activates autoimmune and inflammatory responses.

### No effect, or inhibition, by DPH on arousal

One female respondent (F2) reported no effect of DPH on sexual arousal. F2 was being treated for attention deficit and hyperactivity disorder (ADHD) and anxiety. Another female respondent (F3) reported having high levels of arousal normally which DPH apparently diminished. This was considered a positive effect by the respondent. She had been treated for depression, anxiety, and ADHD, and had experienced early menopause following chemotherapy and was currently treated with tamoxifen.

### Non-sexual side effects

M1 reported tachycardia, anxiety (including paranoia and intrusive thoughts at high doses), visual hallucinations and delirium despite remaining aware. M2 reported occasional urinary hesitancy and memory impairment after ingestion. These were experienced with high doses (450 mg to 500 mg). M2 in particular experimented with other drugs, including stimulants, sedatives, and hallucinogens, and noted that the effect of DPH was similar to that of amphetamines.

## Discussion

The cases described here suggest that DPH-induced sexual arousal is a complex, multi-layered phenomenon involving both pharmacological and psychological dimensions. Respondents reported consistent increases in sexual arousal and genital sensitivity, intensified orgasms, and reinforcement-driven use patterns that appeared independent of the use of other medical or non-medical substances. These findings suggest the existence of a potentially novel arousal mechanism induced by DPH that involves antihistaminergic, anticholinergic, and/or dopaminergic interactions^9^ through a process of disinhibition, or activated by a DPH metabolite, or as part of a rebound effect to the antagonist properties of the drug, as has been reported previously in individuals that experience a stimulant-like effect of the drug. Indeed, the direct pharmacological profile of DPH is not conducive to enhanced sexual function. For example, as noted above, blockade of histamine receptors should prevent the smooth muscle relaxation that allows for genital blood flow. Additionally, muscarinic receptor antagonists are known to inhibit sexual responses in laboratory animals^10^^,^^11^ and to cause erectile dysfunction, decreased sexual desire, and vaginal dryness at high doses in humans.^2^ Lower doses used in the treatment of lower urinary tract symptoms or overactive bladder do not have these effects.^12^

## Limitations

It must be noted that general excitation from DPH is found in a minority of users, so the facilitatory effects reported here are likely limited to this population. Moreover, DPH misuse, especially at high doses, can lead to a number of untoward side effects, including gastrointestinal constipation, cognitive dysfunction, hallucinations, dry eyes, arrhythmias, skin flushing, and urinary retention, effects that have led to calls for DPH to be discontinued.^13^ Although all respondents reported their initial use of DPH as a sleep aid, it is not known if they or others might use it as a self-medication against sexual dysfunction. This should be examined empirically in future studies.

Another limitation involves the potential mechanism of excitation described above. It is not known whether the respondents that described enhanced sexual function were indeed ultrarapid metabolizers with enhanced CYP2D6 enzyme activity. However, it is notable that one respondent, F4, found that DPH was able to override the inhibitory effects of her antidepressant medication on sexual function. This is reminiscent of the ability of the selective dopamine and noradrenaline reuptake blocker bupropion to diminish the inhibitory sexual effects of selective serotonin reuptake blockers,^14^^,^^15^ or the ability of stimulant drugs, like caffeine, dextroamphetamine, ephedrine, methylphenidate, and even sympathetic activation by exercise, to do the same.^16–20^ Ultrarapid metabolizers show increased tolerability of SSRI side effects whereas slower metabolizers show more side effects with greater intensity.^21^ It is possible, therefore, that the sexually enhancing effects of DPH may stem from its metabolism into a compound with stimulant or disinhibitory properties. Future studies of DPH enhanced sexual function should assess CYP2D6 alleles and other potential polymorphisms to determine this possibility. Finally, most respondents were from the U.S., which limits the generalizability of the effects.

## Conclusions

The sexual effects of over-the-counter medications intended for different treatments is an underexplored topic in sexual medicine and pharmacology. Although the antihistamine and anticholinergic actions of DPH can disrupt sexual function and induce a number of untoward side effects at higher doses, in some users, DPH appears to facilitate sexual arousal, genital sensitivity, and pleasure at orgasm. This may occur only in individuals that are ultrarapid metabolizers through the conversion of DPH into a compound with stimulant or disinhibitory properties. More research is required to establish this, along with placebo-controlled studies that examine baseline rates of sexual responding and how they are disrupted or enhanced by DPH. Consistent psychological or medical comorbidities (eg, anxiety, depression) should be examined as well. Finally, recreational use of DPH to enhance sexual or other functions may carry a risk of physical and/or psychological dependence that should be examined critically.
